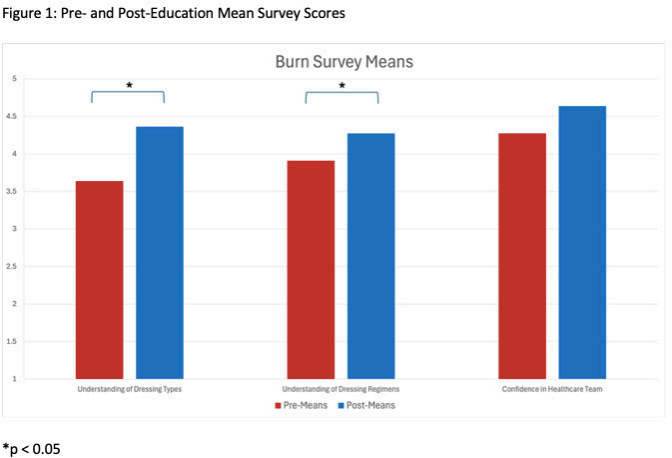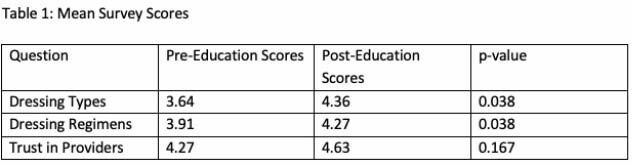# 987 You Put What on Me? Visual Chart Efficacy for Burn Patient Wound Dressing Understanding

**DOI:** 10.1093/jbcr/iraf019.518

**Published:** 2025-04-01

**Authors:** John Austin, Nicole Goldhaber, Parisa Oviedo, Julia Thrasher, Carrie Littlepage, Brian Piatkowski, Rungfa Tangtumnu, Laura Haines, Jeanne lee, Jarrett Santorelli

**Affiliations:** UC San Diego Health Regional Burn Center; UC San Diego Health Regional Burn Center; UC San Diego Health Regional Burn Center; UC San Diego Health Regional Burn Center; UC San Diego Health Regional Burn Center; UC San Diego Health Regional Burn Center; UC San Diego Health Regional Burn Center; UC San Diego Health Regional Burn Center; UC San Diego Health Regional Burn Center; UC San Diego Health Regional Burn Center

## Abstract

**Introduction:**

Acute burn patients frequently undergo complex wound care with a wide variety of dressings, tissue substitutes, and grafts. To improve patient understanding and experience, we piloted a quality improvement program using visual-reference charts to explain graft types and dressing change regimens. We hypothesized the intervention would increase patient understanding and trust in healthcare providers.

**Methods:**

This is a pilot quality improvement study performed at an American Burn Association certified burn center. The most common grafts and dressing regimens were depicted on poster-sized visual reference charts and posted throughout the burn unit. English-speaking, adult patients on the burn service or in the burn clinic were approached for the study. Burn providers educated patients on graft types and dressings using the chart. Before and after education, surveys were performed evaluating patient understanding of burn dressings, their dressing change regimens, and confidence in their healthcare team. Answers were given on a five-point Likert scale. We performed paired t-tests between the pre- and post-education survey results.

**Results:**

Eleven pre- and post-education surveys were completed. After education, patients demonstrated significant increase in their understanding of burn dressings (p = 0.038) and their dressing change regimen (p = 0.038). Patient’s baseline confidence in the healthcare team was high and remained unchanged after education (p = 0.17). Prior to education intervention, only a minority of patients noticed the posters (n = 3, 27.3%) or had someone explain the posters to them (n = 1, 9.1%).

**Conclusions:**

Our study demonstrates that implementing a visual dressing reference can improve patient understanding of their burn dressings and their dressing change regimens. While patients highly trusted their treatment teams, patients were less sure about what their actual burn dressings and regimens entailed. With the help of visual mediums and provider education, we can empower patients with improved knowledge of their burn care, improving their understanding and satisfaction.

**Applicability of Research to Practice:**

Patients benefit with dedicated teaching from visual mediums about their surgical burn dressings. Placing dressing posters throughout burn units will give patients a continued reference. Continuing enrollment in the study will further clarify which populations benefit the most from these teaching sessions. Future studies can look at poster effectiveness in the Spanish-speaking or pediatric populations.

**Funding for the Study:**

N/A